# Multiple freeze-thaw cycles lead to a loss of consistency in poly(A)-enriched RNA sequencing

**DOI:** 10.1186/s12864-021-07381-z

**Published:** 2021-01-21

**Authors:** Benjamin P. Kellman, Hratch M. Baghdassarian, Tiziano Pramparo, Isaac Shamie, Vahid Gazestani, Arjana Begzati, Shangzhong Li, Srinivasa Nalabolu, Sarah Murray, Linda Lopez, Karen Pierce, Eric Courchesne, Nathan E. Lewis

**Affiliations:** 1 Department of Pediatrics, University of California, San Diego, USA; 2 Bioinformatics and Systems Biology Program, University of California San Diego, San Diego, USA; 3 Autism Center of Excellence, Department of Neuroscience, University of California San Diego, San Diego, USA; 4 Department of Medicine, University of California San Diego, San Diego, USA; 5 Department of Bioengineering, University of California San Diego, San Diego, USA; 6 Department of Pathology, University of California San Diego, San Diego, USA; 7 Novo Nordisk Foundation Center for Biosustainability, University of California, San Diego, La Jolla, USA

**Keywords:** RNA-Seq, Quality control, Freeze-thaw, Sample preparation, Differential expression

## Abstract

**Background:**

Both RNA-Seq and sample freeze-thaw are ubiquitous. However, knowledge about the impact of freeze-thaw on downstream analyses is limited. The lack of common quality metrics that are sufficiently sensitive to freeze-thaw and RNA degradation, e.g. the RNA Integrity Score, makes such assessments challenging.

**Results:**

Here we quantify the impact of repeated freeze-thaw cycles on the reliability of RNA-Seq by examining poly(A)-enriched and ribosomal RNA depleted RNA-seq from frozen leukocytes drawn from a toddler Autism cohort. To do so, we estimate the relative noise, or percentage of random counts, separating technical replicates. Using this approach we measured noise associated with RIN and freeze-thaw cycles. As expected, RIN does not fully capture sample degradation due to freeze-thaw. We further examined differential expression results and found that three freeze-thaws should extinguish the differential expression reproducibility of similar experiments. Freeze-thaw also resulted in a 3′ shift in the read coverage distribution along the gene body of poly(A)-enriched samples compared to ribosomal RNA depleted samples, suggesting that library preparation may exacerbate freeze-thaw-induced sample degradation.

**Conclusion:**

The use of poly(A)-enrichment for RNA sequencing is pervasive in library preparation of frozen tissue, and thus, it is important during experimental design and data analysis to consider the impact of repeated freeze-thaw cycles on reproducibility.

**Graphical abstract:**

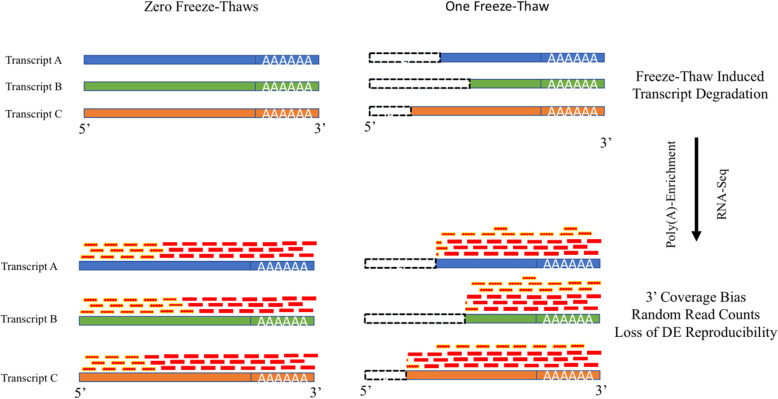

**Supplementary Information:**

The online version contains supplementary material available at 10.1186/s12864-021-07381-z.

## Background

RNA sequencing (RNA-Seq) is a ubiquitous method, used to answer a wide range of biological questions. Methods for aligning, quantifying, normalizing, and analyzing expression data are available through popular packages including Tophat, STAR, cufflinks, SVA, RUV, Combat, DESeq2, edgeR, Kallisto, Salmon, and BWA-MEM [1–11]. Each method aims to accommodate and mitigate the unique challenges presented by RNA-Seq data. Some approaches attempt to account for characterized variability in RNA-Seq measurements due to factors such as sequencing depth, gene length, and transcripts’ physical characteristics (e.g., GC content). Others account for “unwanted variance” due to technical, batch, or experimental variation. In contrast, the influence that sample handling requirements, such as tissue lysis or varying processing times [12–14], have on RNA-seq measurement quality is not comprehensively characterized. Knowledge-gaps in the impact of sample-handling can make it difficult to control for such factors. Adequately characterizing technical variation introduced to RNA-Seq measurements by sample processing steps is important for optimizing sample quality during sample handling, accounting for transcript degradation during data processing, and, consequently, improving the accuracy and reproducibility of sequencing results.

Many steps in sample processing, e.g. sample storage conditions including temperature and the use of stabilitizing reagents, may specifically decrease sample quality by inducing transcript degradation [14, 15]. Non-uniformity in degradation across genes and samples introduces variability in signal and causes inaccurate normalization and transcript quantification [16]. Poly(A)-enrichment methods are commonly used to separate mRNA from other highly abundant RNA molecules (e.g., rRNA, tRNA, snoRNAs, etc.), but variable degradation directly impacts read counts by causing non-uniform transcript coverage [17]. Yet, different sources of RNA degradation can impact RNA-Seq in different manners [18]. Of particular interest, freeze-thaw can induce 20% degradation of spike-in standards per cycle, a factor that may be generalizable to mRNA transcripts [19]. Freeze-thaw cycles increase RNA degradation by disrupting lysosomes which store RNases, freeing the enzymes to promiscuously catalyze nuclease activity [20]. Furthermore, partially defrosted crystals create uneven cleaving pressure on mRNA strands [21, 22]. Despite these observations, the extent to which freeze-thaw negatively impacts count and differential expression in RNA-Seq analyses has not been comprehensively characterized.

Standard sample quality control often relies on RNA integrity number (RIN), which quantifies the 28S to 18S rRNA ratio [23]. RIN-based quality control approaches rely on a heuristic threshold to assess sufficient quality [24, 25]. RIN-based metrics have known confounders such as transcript level, and thus have been called into question as an appropriate quality metric [26]. For example, RIN failed to indicate a decrease in sample quality in lung cancer tissue samples that underwent five freeze-thaw cycles [27] and, in statistical analyses, failed to correct for the effects of degradation [28]. Despite this, many studies rely on RIN to correct for and assess sample quality confounders [18, 29, 30]. This is especially problematic in the case of transcript degradation because RIN scores are assessed by the entire sample, while degradation effects can be transcript-specific [16, 31, 32]. Furthermore, existing studies on degradation are not simply generalizable to freeze-thaw, which has distinct and independent effects on sample quality and must be fully explored as such [18, 33].

Here, we tested the susceptibility of poly(A)-enriched RNA-Seq results after multiple freeze-thaw cycles. We assessed sample quality independently of RIN by simulating read count variability to capture the noise between technical replicates. We found that each additional freeze-thaw cycle increased the random counts between technical replicates by approximately 4%. Subsequently, differential expression reproducibility approached zero after three freeze-thaw cycles. These effects are not captured by RIN. We find that these effects are reflected in increased 3′ bias in read coverage when combining poly(A)-enrichment with freeze-thaw, a phenomenon that appears to be generalizable to publicly available datasets.

## Results

### 3′ bias in read coverage of public datasets is associated with poly(a)-enrichment and freeze-thaw

**Fig. 1:**
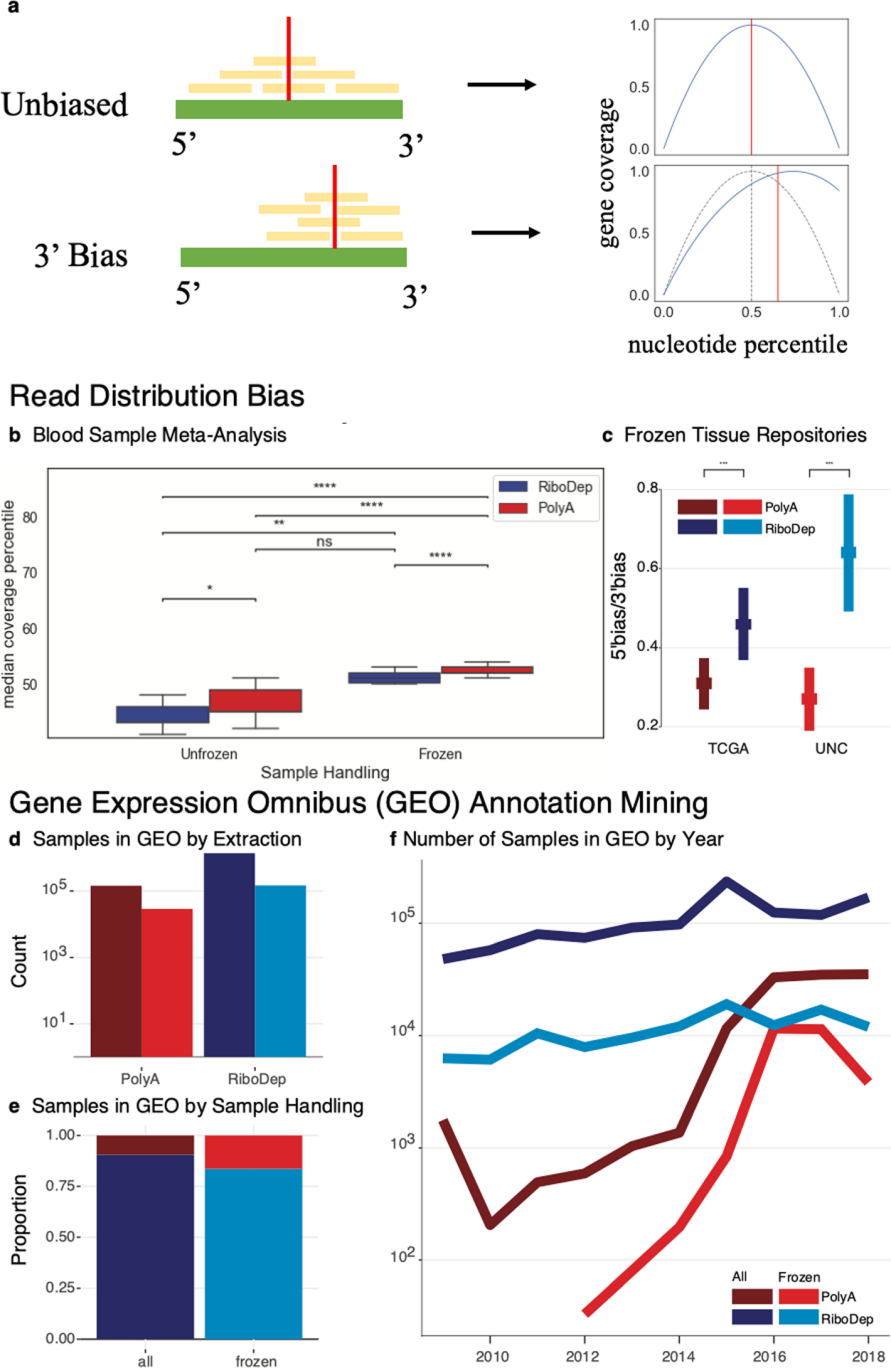
3′ Bias is Exacerbated in Frozen, Poly(A)-enriched Samples Across Multiple Studies. **a** Demonstration for determining median coverage percentile (red vertical line). When coverage is unbiased, reads (yellow) are distributed throughout the entire body of the transcript (green). In the absence of read bias and observing coverage as a function of the nucleotide percentile, we see that cumulative coverage along the transcript reaches 50% half-way through the gene body, at the 50th percentile nucleotide. In contrast, given a 3′ read bias, there is a shift in the distribution of reads and cumulative coverage reaches 50% at, for example, the 60th percentile nucleotide. This can be seen by the "rightward" shift in median coverage percentile towards the 3′ end of the transcript. In the right panel, gene coverage (y-axis) at the i^th^ nucleotide percentile from 5′ to 3′ (x-axis) displayed for the unbiased and 3' biased transcripts. **b** Median coverage percentile was calculated for 237 blood tissue samples spanning 10 RNA-Seq datasets downloaded from SRA. Samples are stratified by sample handling (unfrozen or frozen) and library preparation (poly(A)-enrichment or ribosomal depletion). Read coverage distributions were compared using a two-sided, two-sample t-test with a Benjamini-Hochberg correction (* FDR ≤ 0.05, ** FDR ≤ 0.01, *** FDR ≤ 1e-3, **** FDR ≤ 1e-4). **c** Comparison of 5′ to 3′ bias ratio (y-axis) of samples from the TCGA and UNC tissue repositories (x-axis) between extraction methods (two-sample t-test). Querying human RNA samples listed in GEO from 2008 to 2018, and stratifying by those annotated as “frozen”, we observe (**d**) the number of samples prepared with poly(A)-enrichment or ribosomal depletion (x-axis), (**e**) the proportion of samples extracted using either method, and (**f**) the change in the number of samples over time

We first examined public data to establish initial evidence of the incompatibility of poly(A)-enrichment and frozen samples. Specifically, we analyze the gene-coverage distribution in libraries prepared from frozen samples by either poly(A)-enrichment or ribosomal depletion. Since evidence exists that freeze-thaw enhances transcript degradation, and since poly(A)-enriched samples select mRNA by hybridization to the poly(A)-tail, we expect increased read coverage on the 3′ end of transcripts--3′ bias--when these two factors are combined. To test this expectation, we compared gene body coverage from the 5′ to 3′ end between poly(A)-enriched and ribosomal RNA depleted samples with and without freezing. Specifically, we examined the median coverage percentile, the transcript length-normalized nucleotide percentile at which median cumulative coverage for a given sample is achieved (Fig. 1a).

We compared median coverage percentile between 237 blood-tissue samples spanning 10 publicly available datasets (Supplementary Table S1, Fig. 1b). We found that for either library preparation method, freezing increases 3′ bias (independent t-test, Benjamini-Hochberg correction, FDR ≤ 0.003), but that this increase is much more significant for poly(A)-enriched samples. Additionally, poly(A)-enrichment has a consistently larger 3′ bias than ribosomal depletion (FDR ≤ 0.041). Furthermore, both library preparation and freezing independently and significantly contribute to 3′ bias (two-way ANOVA, *p* ≤ 3.99e-9). In an additional study examining the impact of RNA extraction in frozen tissue from the UNC and TCGA tumor tissue repositories [34], we found a significant (two-sample t-test, *p* < 1e16) decrease in the 5′-to-3′ coverage ratio of poly(A)-enriched samples compared to ribosomal depletion (Fig. 1c). This indicates an increase in 3′ bias of frozen tissues consistent across both repositories.

To determine the breadth of this potential sample processing issue, we explored the prevalence of poly(A)-enrichment from frozen tissue by examining metadata in the Gene Expression Omnibus (GEO). Using GEOmetadb [35], we queried all human RNA samples between 2008 and 2018 extracted with either poly(A)-enriched or ribosomal depletion. There are thousands of samples annotated as “frozen” prepared using either total RNA or poly(A)-enrichment methods (Fig. 1d). In samples annotated as “frozen”, the frequency of poly(A)-enrichment increases from less than 10% to over 25% relative to all samples (Fig. 1e), suggesting that this potentially problematic combination of library preparation and sample storage is prevalent and possibly preferred. Finally, stratifying this trend over time, we see that poly(A)-enrichment, as well as the relative proportion of poly(A)-enriched frozen samples, is increasing in popularity relative to the fairly consistent usage of total RNA extraction (Fig. 1f). Taken together, these results indicate a potential, widespread distortion in RNA-seq associated with a deleterious interaction between poly(A)-enrichment and freeze-thaw. As these results span several studies, each may introduce unaccounted sources of technical variation. To explore this potential more formally, the remainder of our analyses focus on a specific experiment designed to address this question. Specifically, we subjected whole-blood extracted leukocyte samples--with technical replicates--from autistic (ASD) or typically developing (TD) toddlers to a varying number of freeze-thaw cycles, which we record alongside other sample quality metrics such as RIN.

### An additional freeze-thaw cycle increases random read counts 1.4-fold

To address the scarcity of analyses on the effect of freeze-thaw on RNA-seq measurements, we use our technical replicates to compare changes in sample quality across freeze-thaw cycles. We first note that neither RIN nor TIN capture significant (one-sided Wilcoxon test) decreases in sample quality due to increased freeze-thaw (Fig. S1). Given previous indications that these metrics may not sufficiently address transcript degradation [16, 26–28], we instead measure the introduction of noise to samples (Fig. S2-3). We define noise as the fraction of reads in a sample that are randomly counted, rather than mapping to a sample-specific gene. To estimate noise, we simulated the randomness in read counts between technical replicates (Supplementary Methods). By comparing technical replicates that have undergone the same number of freeze-thaws, we can calculate the expected noise in a sample at a given number of freeze-thaw cycles. Since noise does not rely on RIN, we can compare freeze-thaw and RIN effects independently.

**Fig. 2:**
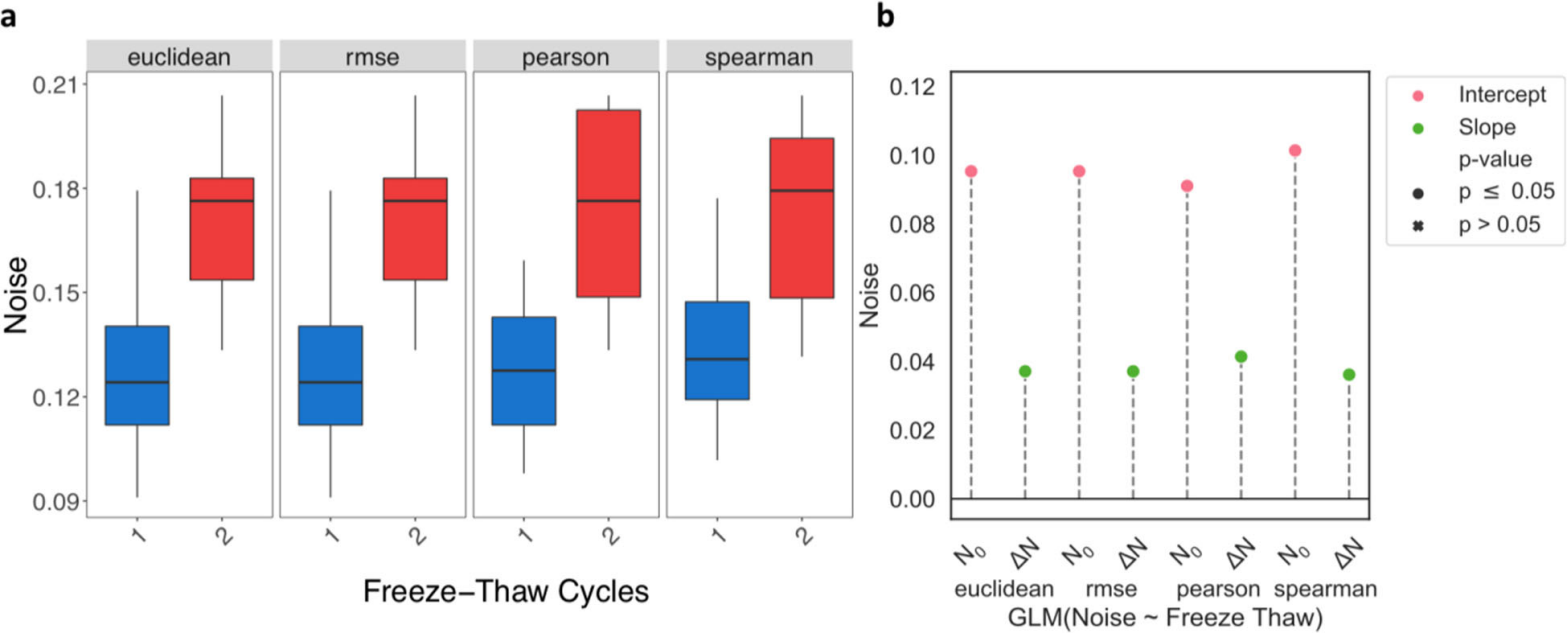
Higher Noise in Samples with More Freeze-Thaw Cycles. From left to right, noise—the randomness in read counts between technical replicates—is estimated using Euclidean distance, RMSE, Pearson correlation, and Spearman correlation. **a** Box plots of noise for samples that underwent either one or two freeze-thaws. **b** A linear regression was used to determine the expected noise without freeze-thaw (N_0_, pink) and the expected change in noise with each additional freeze-thaw (ΔN, green). All estimates are significant (p ≤ 0.05)

Median noise increased 1.4-fold from one to two freeze-thaw cycles (one-sided Mann-Whitney U test, *p* ≤0.007) on average across all measures (Fig. 2a). By definition, technical replicates reveal variation due to technical measurement error. We estimated noise between technical replicates that have not undergone freeze-thaw to range between 9.11–10.15% (Wald test, *p* ≤5.77e-7). The expected increase in noise per additional freeze-thaw cycle was estimated to be 3.6–4.1 percentage points (Wald test, *p* ≤8.12e-3) (Fig. 2b). The introduction of random reads to samples by freeze-thaw cycles may have substantial effects on count quantification (see Discussion) and, consequently, downstream analyses such as differential expression.

### RIN does not predict additional noise after one freeze-thaw cycle

Next, we asked whether our observations that RIN does not sufficiently capture changes in sample quality due to freeze-thaw (Fig. S1) could be extended to noise. Specifically, we tested whether RIN can reflect the differences in sample quality as measured by noise.

**Fig. 3:**
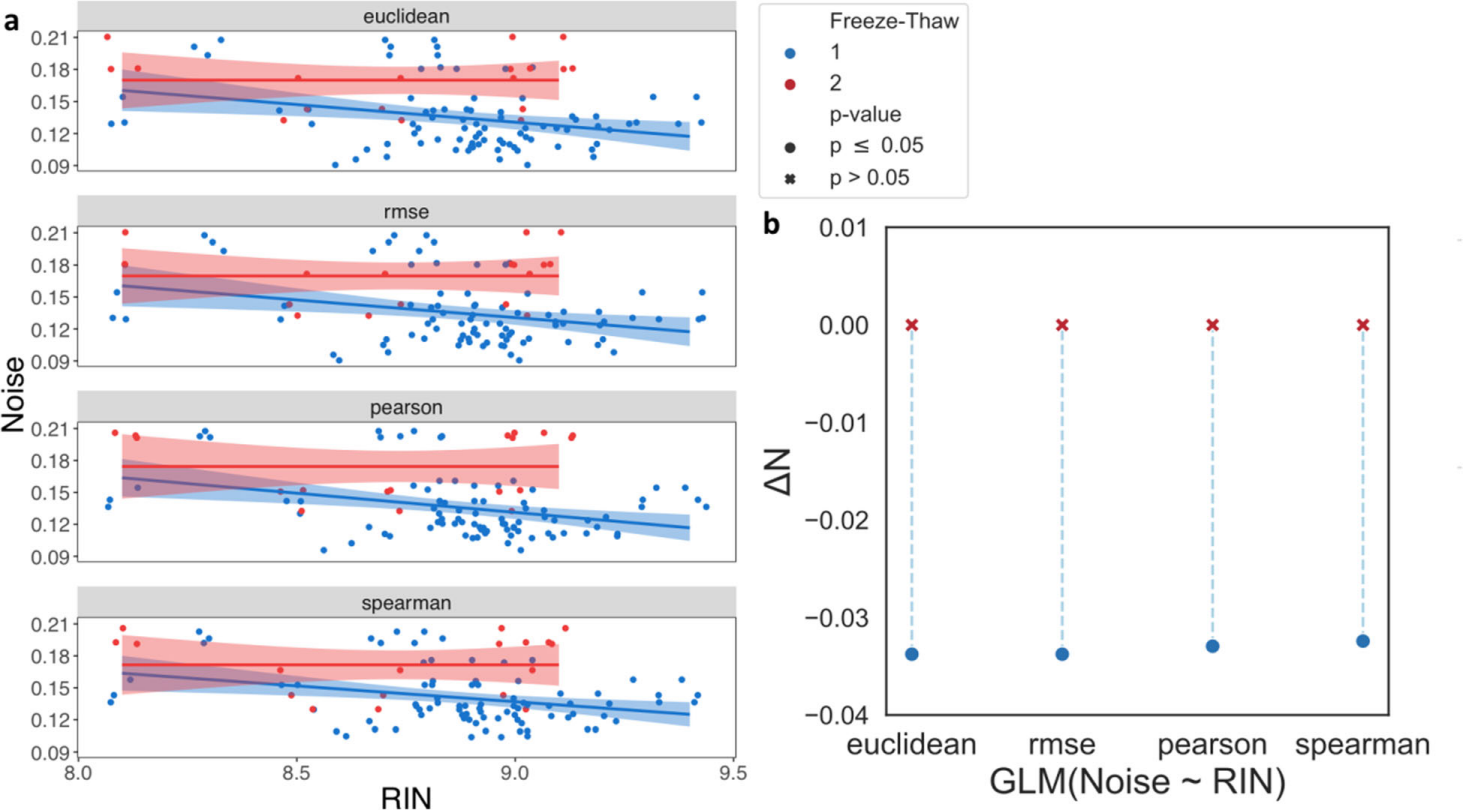
Discrepancy in the Relationship Between Noise and RIN due to additional Freeze-Thaw. Examining the relationship between noise, calculated by Euclidean distance, RMSE, Pearson, and Spearman correlation, for samples that underwent either one (blue) or two (red) freeze-thaw cycles. **a** Scatter plots comparing noise (y-axis) to RIN (x-axis). The solid lines show a linear regression fit and the shaded regions show the 95% confidence interval for this fit. **b** The expected change in noise due to a one-point increase in RIN (ΔN, y-axis) estimated by linear regressions. Significant estimates (p ≤ 0.05) are marked by a circle and insignificant estimates are marked by a cross

When only considering samples that underwent one freeze-thaw, each unit increase in RIN decreases noise by 3.24–3.38 percentage points for all metrics (Wald test, *p* ≤6.3e-3) (Fig. 3a-b, Fig. S14). Yet, when only accounting for samples that underwent two freeze-thaw cycles, noise does not significantly change as RIN increases. Taken together, these results indicate that while RIN can be a good measure of noise for samples that underwent one freeze-thaw, it does not capture the loss in sample quality induced by two freeze-thaw cycles.

### Differential expression similarity increases 10.3% in high quality samples

Next, we investigated how the introduction of noise impacts differential expression (DE) analysis. We assessed DE reproducibility by generating thousands of sample combinations, i.e. subsets, with varying sample quality (Fig. S4). We define sample quality by the aggregate number of freeze-thaw cycles or RIN. We ran DE across ASD-TD groups and compared results between subsets of various sizes (4–14 samples). We measure reproducibility using similarity or discordance, based on correlation and dispersion, respectively; higher similarity and lower discordance each represent higher reproducibility. We use these measures to assess differences that arise between subsets consisting of high quality (low freeze-thaw or high RIN) and low quality (high freeze-thaw or low RIN) samples.

We held two expectations regarding the effect of sample quality on DE reproducibility in the context of similarity: 1) the reproducibility between subsets with high quality samples should be higher than those with low quality samples at any given subset size, and 2) subset size and sample quality should interact to increase the reproducibility of DE analysis; this would be reflected by a higher rate of increase in reproducibility with respect to subset size for higher quality subsets.

**Fig. 4:**
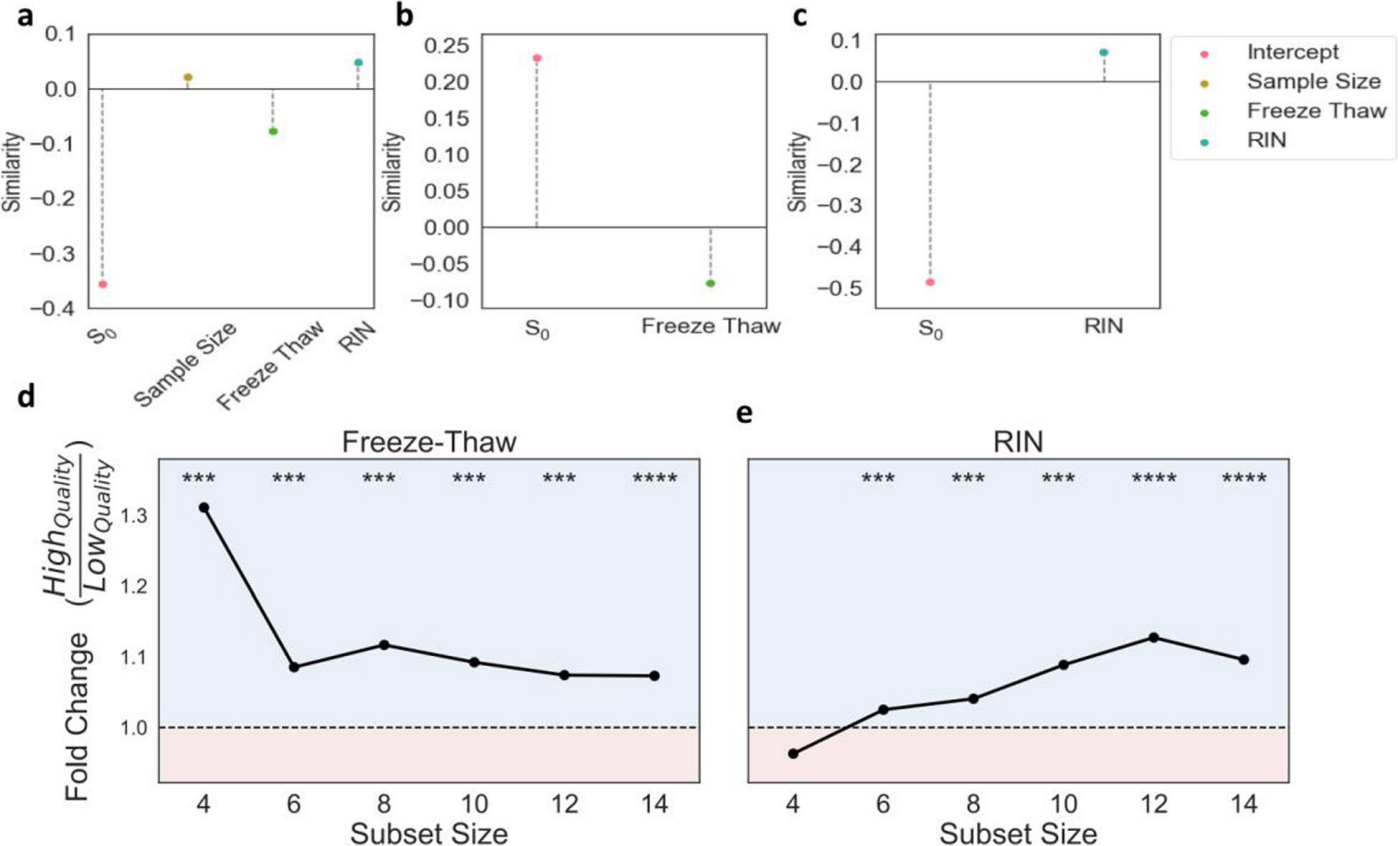
Freeze-Thaw and RIN Both Demonstrate Higher Similarity with Increased Quality. Top panels summarize results of linear regressions used to quantify the change in similarity per unit increase in (**a**) sample size, number of freeze-thaws and RIN combined additively, (**b**) only the number of freeze-thaws, and (**c**) only RIN. S_0_ represents the intercept estimate and sample size, freeze-thaw, and RIN represent coefficient estimates. All estimates are significant. Bottom panels demonstrate fold-change in median similarity of high quality subsets with respect to low quality subsets at each subset size. The region shaded in blue (fold-change > 1) indicates instances where the median similarity for high quality is larger than that of low quality. The region shaded in red (fold-change < 1) indicates instances where the median similarity for low quality is larger than that of high quality. Average (**d**) freeze-thaw or (**e**) RIN are used to place subset pairs into high or low quality sample bins. Significance (one-sided Mann-Whitney U test) of comparisons in similarity distributions between high and low quality subset pairs are displayed above each subset size

As expected, similarity increases with subset size (Fig. S10), as reflected by the estimated 0.02 (Wald test, *p* = 2.2e-5) increase in similarity per additional sample (Fig. 4a**)**; thus, expected similarity would increase by 0.20 in a subset with 14 samples relative to a subset with 4 samples. Regression results for each model predicting similarity are reported in Supplementary Table S5.

To measure similarity, we took the pairwise Spearman correlation of the log-fold change values between subsets. We tested our first expectation by placing subset pairs into high and low sample quality bins--defined by either RIN or freeze-thaw--for each subset size and comparing their similarity values. Regardless of sample quality, DE similarity increases with subset size. Yet, for nearly all subset sizes, higher quality bins have significantly (one-sided Mann-Whitney U test, *p* ≤ 2.8e-17) higher similarity than low quality bins (Fig. 4d-e). Across subset sizes, we observed an average 1.13-fold and 1.06-fold increase in similarity from low to high quality samples for freeze-thaw and RIN, respectively.

Similarity significantly (Wald test, *p* ≤ 9.2e-3) decreases with the number of freeze-thaw cycles and increases with RIN when accounting for the effects of sample size (Fig. 4a-c), validating our second expectation. Similarity decreases by 0.077 per additional freeze-thaw cycle (Wald test, *p* = 8.77e-4). Given the estimated similarity of 0.23 for samples that have not undergone freeze-thaw, this implies that DE reproducibility will approach zero after approximately three freeze-thaw cycles (Fig. 4b). Even when accounting for subset size and the effects of RIN, the estimated decrease in similarity from freeze-thaw is nearly the same--0.078 (Wald test, *p* = 8.77e-4); this further corroborates that RIN alone cannot capture the changes in sample quality due to freeze-thaw. Taken together, these results indicate that higher sample quality increases DE reproducibility as measured by similarity.

### Discordance decreases nearly 5-fold in high quality samples

We further investigated the relationship between DE reproducibility and sample quality using an effect size sensitive measure of discordance (Fig. S9). Specifically, we explored how sample quality affects the relationship between discordance and the DE effect size--measured by the mean-variance standardized effect--at each subset size. In this context, we expected 1) discordance at any given effect size to be lower in high-quality subsets and 2) the rate of increase in discordance to be lower in high quality subsets relative to low quality subsets.

Corresponding to the regression models used for this analysis, we label the expected change in discordance per unit increase in fold-change effect size as ΔD. We observed a significant (Wald test, *p* ≤ 9.45e-141) decreasing trend in ΔD with increasing subset size (Fig. S11, Supplementary Table S6).

**Fig. 5:**
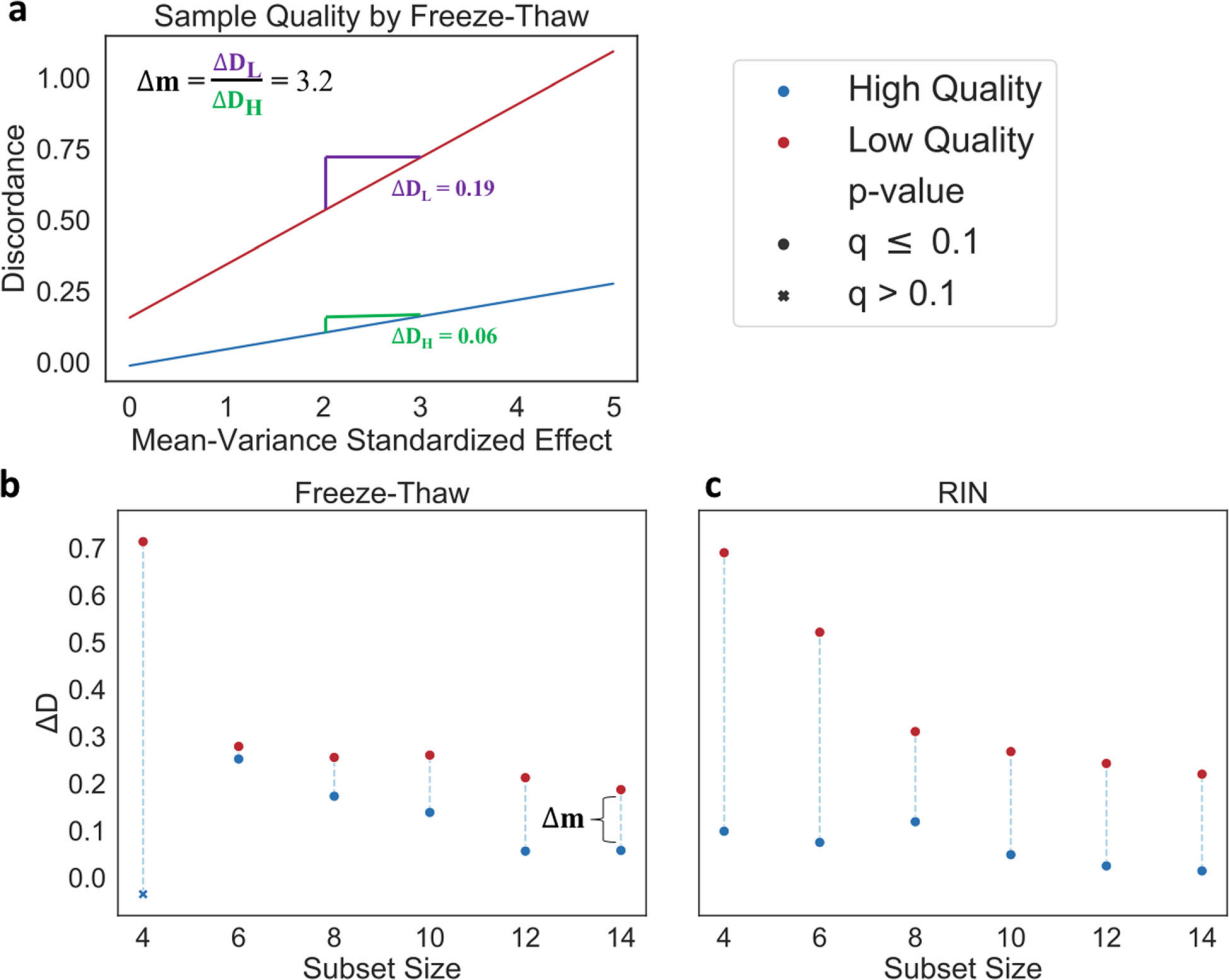
Higher Sample Quality has Lower Discordance at Each Subset Size. Linear regression estimates of discordance predicted from effect size and subset quality for each subset size. High sample quality (blue) is compared to low sample quality (red). ΔD values represent the change in discordance per unit increase in effect size. **a** The predicted discordance with respect to the mean-variance standardized effect at a subset size of 14; sample quality is assessed by freeze-thaw. The disparity (Δm) between the change in discordance per unit increase in effect size for high (D_H_) and low (D_H_) quality subsets is also displayed. Summary of results for each subset size (x-axis) for sample quality represented by either (**b**) freeze-thaw or (**c**) RIN. Significant estimates (Wald test, Benjamini-Hochberg FDR correction, q ≤ 0.1) are marked by a circle and insignificant estimates are marked by a cross. For (**b**) freeze-thaw, Δm corresponding to panel (**a**) is also displayed

We estimate discordance with respect to effect size at each subset size and for subsets of either high or low quality. As expected, independent of sample quality, ΔD demonstrates an overall decreasing trend with respect to subset size for both RIN and freeze-thaw. With respect to freeze-thaw, at a subset size of 6, there is a 1.1-fold decrease in the value of ΔD from low quality subsets to high quality subsets. The disparity in ΔD between high and low sample quality (Δm = ΔD_Low Quality_ / ΔD_High Quality_) increases nearly monotonically through to the subset size of 14, at which point there is a 3.2-fold decrease (Fig. 5a). This monotonicity indicates that the observed relationship between discordance and sample quality is consistent. Furthermore, it causes notable differences in discordance values, even at low effect sizes.

Consistent with our expectations, ΔD is lower for high quality subsets as compared to low quality subsets for both freeze-thaw and RIN across all subset sizes (Fig. 5b-c). Nearly all estimates are significant after multiple test correction (Wald test, Benjamini-Hochberg FDR correction, q ≤ 0.07), with the exception of those for the smallest subset size for freeze-thaw.

Taken together, these results indicate that higher sample quality increases DE reproducibility as measured by discordance.

### Additional freeze-thaw cycles show increased 3′ bias in poly(a)-enriched but not ribosomal RNA depleted samples

Finally, we asked whether repeated freeze-thaw cycles can induce a 3′ bias, consistent with the induction of random reads and the loss of DE reproducibility as well as our initial observation in the public datasets.

Using the median coverage percentile, we found a shift in mRNA coverage towards the 3′ end of the poly(A)-enriched samples relative to ribosomal depletion (Fig. S13a). Specifically, the median coverage percentile for poly(A)-enriched samples is significantly (one-sided Wilcoxon test, *p* < 2.2e-16) larger than that of ribosomal RNA depleted samples (Fig. S13b). Samples prepared with poly(A)-enrichment have more 3’bias compared to ribosomal depletion in both one (one-sided Wilcoxon test, *p* = 7e-15) and two (one-sided Wilcoxon test, *p* = 5.9e-5) freeze-thaw cycles (Fig. S13d). Altogether, this indicates an overall 3′ bias of poly(A)-enriched samples, even independently of freeze-thaw (Fig. S13b).

**Fig. 6:**
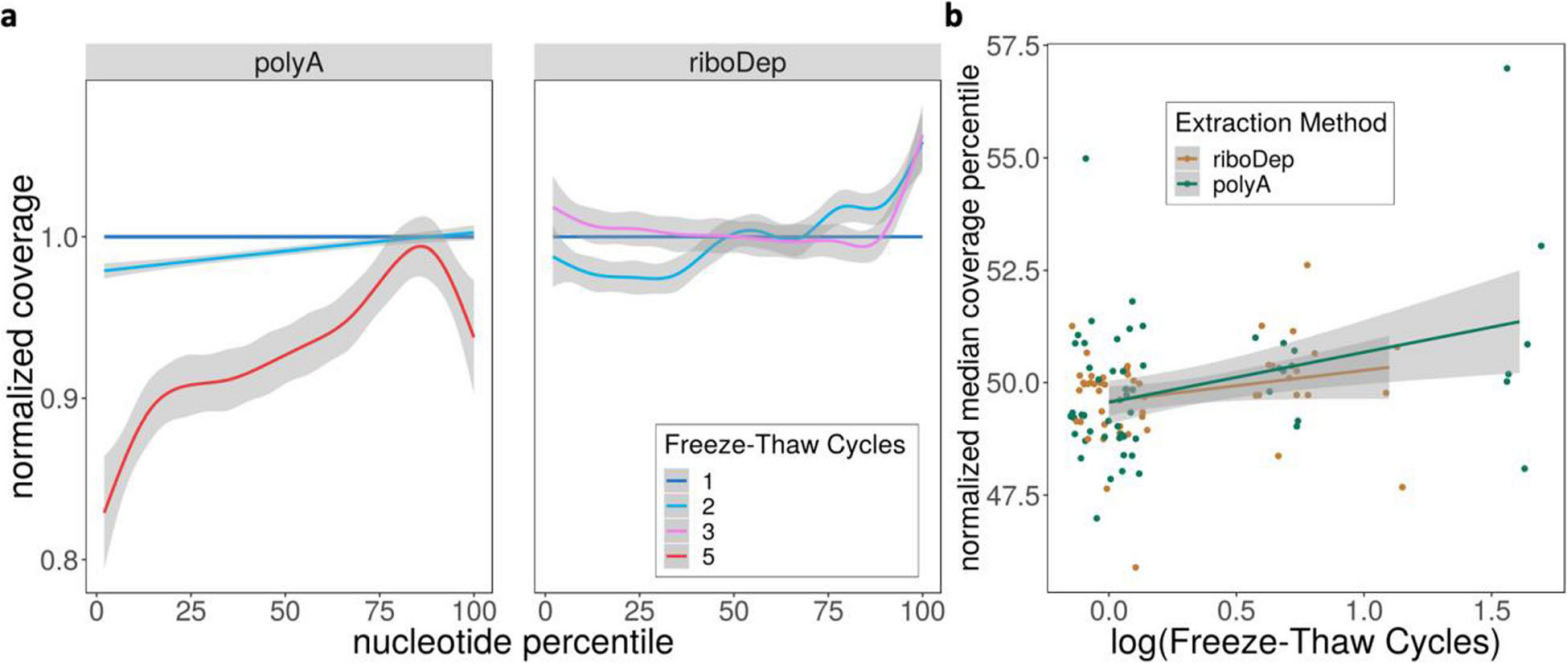
Freeze-Thaw Cycles Exacerbate 3′ Bias in poly(A)-enriched samples. **a** Gene coverage (y-axis) at the i^th^ nucleotide percentile (x-axis) for samples that underwent 1–5 freeze-thaws and were extracted using either poly(A)-enrichment or ribosomal depletion. Coverage is normalized to samples that underwent one freeze-thaw. For each sample, coverage is averaged across all genes; samples are aggregated using generalized additive model smoothing, with shaded regions representing 95% confidence intervals. **b** Linear model fits comparing the change in normalized median coverage percentile to the number of freeze-thaw cycles (log-transformed) for ribosomal depletion (orange) or poly(A)-enrichment (green)

Crucially, this 3′ bias is accentuated when samples are stratified by the number of freeze-thaw cycles (Fig. 6a). We observe a significant increase (Wald test, *p* = 0.007) in normalized median coverage percentile due to the number of freeze-thaw cycles in poly(A)-enrichment. The increase was not maintained in ribosomal RNA depleted samples (Wald test, *p* = 0.07) (Fig. 6b, Supplementary Table S8). For poly(A)-enriched samples, normalized median coverage percentile increases 1.12 percentage points per log freeze-thaw cycle; freeze-thaw cycles were log-transformed to stabilize variance. We further demonstrate a dependency of 3′ bias on freeze-thaw cycles by showing that median coverage percentile significantly increases with freeze-thaw in poly(A)-enriched samples (Kruskal-Wallis test, *p* = 0.041). This 3′ bias is particularly apparent after five freeze-thaw cycles (one-sided Wilcoxon test, *p* = 0.008). Unlike poly(A)-enrichment, ribosomal depletion, while demonstrating significant differences in median coverage percentile between freeze-thaws (Kruskal-Wallis test, *p* = 0.012), does not follow a trend due to increases in freeze-thaw cycles. This is highlighted by the fact that the difference in median coverage percentile between one and two freeze-thaw cycles is significant (one-sided Wilcoxon-test, *p* = 0.001), but the remaining comparisons are not (Fig. S13c).

Taken together, these analyses indicate that poly(A)-enrichment inherently introduces a 3′ bias in coverage as compared to ribosomal depletion, and that this bias is exclusively exacerbated in poly(A)-enriched samples due to freeze-thaw cycles. Thus, 3′ bias may indicate the severity of freeze-thaw induced signal degradation in poly(A)-enriched samples. If this 3′ bias is the root cause of freeze-thaw induced instability in absolute and differential RNA-seq quantification, such instabilities may be subverted by substituting poly(A)-enrichment for ribosomal depletion during library preparation.

## Discussion

Despite the utility and ubiquity of RNA-Seq, many of the confounding elements associated with the technology are still being characterized. In this work, we demonstrated how one such confounder--freeze-thaw--impacts sample quality and downstream analyses. We highlighted biases in publicly available datasets, and observed an increased 3′ bias in read coverage distributions when both freeze-thaw and poly(A)-enrichment are combined. Proceeding with RNA-seq from frozen leukocytes drawn from a toddler Autism cohort, we first measured the noise between technical replicates. This allowed us to examine the impact of freeze-thaw cycles and the ability of RIN to capture those impacts. Next, we examined the impact of freeze-thaw cycles on the robustness and reproducibility of differential expression analysis. By our estimates and at these subset sizes, DE reproducibility approaches zero after three freeze-thaw cycles (Supplementary Table S5). Finally, we demonstrated that poly(A)-enriched samples demonstrate substantial 3′ bias in read coverage with increased freeze-thaw cycles. Our results have implications with regards to technical variation due to sample handling, the sensitivity of differential gene expression analysis for frozen tissues and samples, and the utility of RIN.

Technical variation in RNA-Seq is substantial and can be attributed to a variety of factors, including read coverage, mRNA sampling fraction, library preparation batch, GC content, and sample handling [36, 37]. As such, accounting for technical variation has been a major research area of focus for the past decade [1, 2, 5, 37, 38]. Degradation in combination with poly(A)-enrichment is a known source of variation in RNA-Seq. Yet, before technical variation can be accounted for, it must be characterized. While studies have looked into the effect of degradation on RNA-Seq, each mode of degradation impacts sample quality differently, and direct connections between freeze-thaw and sample quality has mainly been assessed via RIN [18, 39, 40].

Our noise estimates help delineate technical variation due to freeze-thaw and may be more sensitive than RIN. Furthermore, the resulting noise provides an estimate for the number of random read counts associated with a gene. For example, given an average 25 million reads sequenced per sample, our approximate 4 percentage points increase in noise per freeze-thaw cycle (Fig. 2b) yields an expected randomness in 1 million reads per sample. Approximating the number of protein-coding genes in the human genome to be 20–25 thousand [41], we can expect a difference of ~ 40–50 additional random counts per gene to exist between technical replicates due to a freeze-thaw cycle (Supplementary Methods, Fig. S15). Thus, each freeze-thaw cycle introduces a non-negligible level of noise to the quantification of gene expression and differential expression of such genes.

To check for the possibility that there is a signature which can help correct for freeze-thaw distortion of RNA-Seq, we attempt to find a group of consistently differentially expressed genes due to freeze-thaw. We find no such signature (Supplementary Results, Supplementary Tables S9-10). This is expected, given that a major source of reduced sample quality due to freeze-thaw is mRNA degradation, which occurs randomly for each transcript and sample. A possible path forward is to correct for sample degradation. Several methods have been proposed for this. While some of these methods rely on RIN or similar metrics (e.g. mRIN, TIN, etc.) [18, 42], others have implemented statistical frameworks which account for gene-specific biases. DegNorm, for example, accounts for the gene-specific relative randomness in degradation in its correction approach [16]. Quality surrogate variable analysis (qSVA) specifically improves differential expression by identifying transcript features associated with RNA degradation [28]. Furthermore, there are recent methods which only assay the 3′ end of a transcript and therefore claim robustness in degraded samples [43].

The effect of freeze-thaw and resultant degradation on RNA-Seq is particularly concerning when considering differential gene expression analysis. It has been observed that RNA degradation can induce the apparent differential expression in as many as 56% of genes [42]. To this end, we quantified this loss of DE reproducibility by measuring similarity and discordance in the context of sample quality. We found a decrease in reproducibility with both decreasing RIN and increasing freeze-thaw. Interestingly, for most reproducibility assessments, we observed a monotonic or near monotonic increase in disparity between low and high quality subsets with respect to subset size. Similarity demonstrated a larger average magnitude of disparity for freeze-thaw, whereas discordance demonstrated a larger average magnitude of disparity for RIN.

Based on our analysis, the utility of RIN in assessing quality when samples undergo freeze-thaw is questionable. The non-uniformity in mRNA degradation [44–47] due to freeze-thaw sheds light on these challenges, since RIN cannot quantify quality at the individual gene level [23]. This is reflected in the fact that samples with RIN > 8 demonstrate degradation [32]. Furthermore, results assessing the effect of freeze-thaw cycles on RIN are inconclusive. While some studies claim RIN can be used to account for degradation effects in RNA-Seq [18], others suggest it does not sufficiently capture the effects of degradation on sample quality [26, 28]. When directly observing the effect of freeze-thaw on RIN, studies have found a negligible effect [12] or can only detect an effect after numerous cycles [27, 48].

As such, we re-examined the utility of RIN as a measure of sample quality in relation to our noise estimation of random reads per sample [23]. We found that while noise increases with both decreasing RIN and increasing freeze-thaw, RIN may be an insufficient indicator of quality for samples that have undergone two or more freeze-thaws. Given these results, RIN may not always be a good metric to quantify the difference between technical replicates that have undergone variable sample handling [16, 26–28]. We validate noise by confirming that it does not change with input RNA concentration, excepting outliers (Fig. S12). Therefore, noise could be a useful supplement to RIN when technical replicates are present.

The fact that our predicted decrease in similarity due to freeze-thaw does not change when incorporating RIN into our model further indicates that RIN alone cannot capture the changes in sample quality due to freeze-thaw. Despite this, RIN is a good indicator of sample quality, if not specifically for freeze-thaw. This is reflected in the fact that RIN validates our expectations for DE reproducibility analysis and the comparable range of noise, similarity, and discordance values between freeze-thaw and RIN assessments.

Finally, to confirm our expectation that freeze-thaw decreases sample quality [17, 19–22] and to further characterize the underlying mechanism, we validated the presence of a 3′ bias in coverage. This builds on our and others’ observations that a lower percentage of poly(A)-enriched transcripts are covered [40]. We compared coverage to ribosomal RNA depleted RNA-Seq data, which does not use 3′ hybridization to retain transcripts. We find that poly(A)-enrichment does in fact introduce a strong 3′ bias in coverage as compared to ribosomal depletion. This bias is further exacerbated with additional freeze-thaw cycles in poly(A)-enriched but not ribosomal RNA depleted samples. This implies that degradation due to freeze-thaw does not impact RNA-sequencing of ribosomal RNA depleted samples to the extent that it does in poly(A)-enriched samples. In light of our demonstrations that 3′ bias is associated with a substantial increase in noise and a decrease in DE reproducibility, these findings suggest that RNA-seq from samples that have both been poly(A)-enriched and undergone freeze-thaw cycles likely has unknown, diminished stability. While not all studies have technical replicates to estimate noise, the presence of exaggerated 3′ bias when poly(A)-enrichment is combined with freeze-thaw can be a simple indicator of RNA-seq distortion.

## Conclusion

Altogether, these results indicate that transcriptomics quality control steps cannot rely on RIN alone for samples that have undergone poly(A)-enrichment and multiple freeze-thaws. Furthermore, accounting for the effect of freeze-thaw on poly(A)-enriched RNA sequencing is crucial. Poly(A)-enrichment is prevalent for RNA-sequencing, and, in parallel, samples that undergo multiple freeze-thaws are common in many protocols, especially rare tissues, e.g., postmortem neural tissue. Yet, there is no clear recommendation to avoid poly(A)-enrichment following multiple freeze-thaws. Our results indicate that ribosomal depletion could be a better alternative when freeze-thaw is necessary.

## Methods

Terminology used throughout the paper and described in the proceeding methods sections is summarized in Table 1.

**Table 1:** Various terms used in assessing the effect of freeze-thaw on RNA-sequencing, their definitions, and the specific analyses they are applied to

Category	Term	Definition	Analyses
RNA sequencing	Distortion	A generic term referring to changes in RNA-sequencing data introduced due to technical factors.	
	Consistency	A generic term referring to the reproducibility of RNA-sequencing results between samples.	
Sample Quality	Noise (randomness)	The fraction of reads in a sample that are randomly counted, rather than mapping to a sample-specific gene.	Results section 2 and 3
	Freeze-thaw	The number of freeze-thaw cycles a sample undergoes. A freeze-thaw cycle is defined as freezing a sample in − 80 °C for at least 24 h, proceeded by thawing it to room temperature, with the first hour spent on ice.	All results sections
	RIN	The RNA integrity number as previously decsibed [23]	All results sections
DE Reproducibility	Similarity	Spearman correlation of LFC results from differential expression on sample subsets. Correlation was taken between all pairs of subsets.	Results section 4 and 5
	Discordance	Standard deviation of LFC results from differential expression on sample subsets. Standard deviation was taken across all subsets for each gene.	Results section 4 and 5
Bias	3′ Bias	The extent to which reads map in a skewed manner to the 3′ end of a transcript.	Results section 1 and 6
	Median coverage percentile	The nucleotide percentile (relative to transcript length) at which median cumulative coverage across a transcript is achieved; cumulative coverage is aggregated from the 5′ end to the 3′ end. This is a measure of bias in which a larger median coverage percentile indicates more 3′ bias and vice versa	Results section 1 and 6

### Sample collection and storage

Blood samples drawn from male toddlers with the age range of 1–4 years were usually taken at the end of the clinical evaluation sessions. To monitor health status, the temperature of each toddler was monitored using an ear digital thermometer immediately preceding the blood draw. The blood draw was scheduled for a different day when the temperature was higher than 37 °C. Moreover, blood draw was not taken if a toddler had some illness (for example, cold or flu), as observed by us or stated by parents. We collected 4–6 ml blood into EDTA-coated tubes from each toddler. Blood leukocytes were captured using LeukoLOCK filters (Ambion). After rinsing the LeukoLOCK filters with PBS, the filters were flushed with RNAlater (Invitrogen) to stabilize RNA within the intact leukocytes. After RNA stabilization, the LeukoLOCK filters were immediately placed in a − 20 °C freezer. Additional RNA standards were sourced from normal human peripheral leukocytes pooled from 39 Asian individuals, ages 18 to 47 (Takara/ClonTech: 636592). The RNA standards underwent 1–5 simulated freeze-thaw cycles; a freeze-thaw cycle is defined as freezing a sample in − 80 °C for at least 24 h, proceeded by thawing it to room temperature, with the first hour spent on ice.

### RNA extraction, sequencing and quantification

For 47 samples (from 16 individuals), mRNA was extracted using polyA selection with the TruSeq Stranded mRNA library preparation kit (Illumina). Ribosomal depletion was used to prepare an additional 52 samples. Relevant metadata regarding poly(A)-enriched and ribosomal depleted samples can be found in Supplementary Table S2-3. Ribosomal RNA depleted samples used the TruSeq Stranded Total RNA with RiboZero Gold library preparation kit (Illumina). RNA Integrity Numbers (RIN) were measured using a NanoDrop ND-1000 (ThermoFisher). Both library preparation kits use random hexamers for first-strand cDNA synthesis, improving the accuracy of comparisons across isolation methods and potentially mitigating 3′ bias due to priming methods [49]. Poly-A selected samples were sequenced using 50-base pair single end sequencing on a HiSeq4000 (Illumina) to a depth of 25 M reads. The ribo-depletion prepared libraries were sequenced using 100-base pair paired end sequencing on a HiSeq4000 (Illumina) to a depth of 50 M reads.

Fastq files for each sample underwent quality control using FastQC (v0.33). PolyA and adaptor-trimming were conducted using Trimmomatic [50]. Reads were aligned to the gencode annotated (v25) human reference genome (GRCh38) using STAR (v2.4.0) [7]. Alignments were processed to sorted SAM files using SAMtools (v1.7) [51]. Finally, HTSeq (v0.6.1) was used to quantify reads [51, 52].

### Estimation of noise between technical replicates

To estimate noise between technical replicates of the same individual blood samples, we simulate random loss and gain of reads (Fig. S2). Unlike other metrics, e.g. Euclidean distance, “noise” allows us to quantify the dissimilarity between samples at the scale of raw counts. One technical replicate was chosen as the “reference” replicate, making the other technical replicate the “target” replicate. To measure noise at a given number of freeze-thaw cycles, we only compared technical replicates that had undergone the same number of freeze-thaw cycles. The dissimilarity between replicates is measured by one of four metrics (Euclidean distance, RMSE, Pearson correlation, and Spearman correlation). We iteratively add and remove random reads to the reference replicate until the dissimilarity between the simulated replicate and the reference replicate was equal to the dissimilarity between a target replicate and the reference replicate (Fig S2, Fig. S3). We define the noise between the reference and target replicate as the fraction of reads added or removed per total reads in the reference replicate to achieve the aforementioned level of dissimilarity. We represent this as a percentage, e.g. 5% noise between a reference and target replicate can be interpreted as 5% randomness between their reads. For additional details on noise simulation, see Supplementary Methods.

### Measuring the effect of sample quality on noise

Unless otherwise specified, all linear regressions in all analyses were performed using a generalized linear model (GLM) with an identity link function.

To measure the association between noise and sample quality metrics (number of freeze-thaw cycles, input RNA concentrations, and RNA integrity number), we used a linear regression. The significance of the model parameters is determined by the Wald test. All results are reported in Supplementary Table S4.

For each model, to mitigate the contribution of potential confounding variables, samples with input RNA concentrations in the top and bottom 5% (|z| ≥ 1.645) were removed, decreasing the total number of samples from 47 to 41. For noise prediction from concentration, samples with more than one freeze-thaw were also excluded, decreasing the total number of samples to 35. Noise prediction from the RNA integrity number (RIN) was run separately for samples that had undergone one freeze-thaw and samples that had undergone two freeze-thaws.

### Differential expression analysis

We assess whether the observed sample qualities (measured by number of freeze-thaw cycles and RIN) have an impact on differential expression (DE) reproducibility using a resampling approach. DE was run on random subsets of varying sample sizes (Fig. S4). Before subsetting, we filtered our expression matrix for genes with an average count ≤20 across all samples. This reduced the number of genes from 10,028 to 4520. The total number of samples considered was 46 when disregarding samples that were industry standards, were not assigned to either an autism-spectrum disorder (ASD) or typically-developing (TD) indication, or did not have a recorded sample quality value.

We generated subsets containing *N* = 4–14 samples. For each subset size N, we generated 2000 unique subsets. Each subset had an equal number of TD or ASD samples. Additionally, only one replicate from each blood sample could be included. These requirements limited our subset size to a maximum of 14 samples.

DE between ASD and TD subjects was conducted using DESeq2 (v1.20.0) [1]. Fig. S10 summarizes DE results for all subsets. To account for potential confounders, we used RUV to introduce a control covariate to our design matrix (RUVSeq v1.14.0) [5]. Specifically, we use a set of “in-silico empirical” negative control genes, including all but the top 5000 differentially expressed genes as described in section 2.4 of the documentation for RUVseq (http://bioconductor.org/packages/release/bioc/vignettes/RUVSeq/inst/doc/RUVSeq.pdf). We confirm that RUV produces consistent results with previous Autism leukocyte gene expression signatures [53, 54] (see Supplementary Results).

### Similarity to assess differential expression reproducibility

To assess DE reproducibility, we measure the similarity in log-fold-change (LFC) values between DE runs. Similarity is calculated as the Spearman correlation in the LFC between a pair of subsets of the same size (Fig. S6); we measured similarity in a pairwise manner between all subsets of the same size. Genes with a median base mean (the mean of counts of all samples, normalizing for sequencing depth) or median LFC in the bottom 10th percentile across all subsets were excluded to filter for low magnitude effects (Fig. S5).

Average RIN and freeze-thaw were measured for all subset pairs. Resulting distributions for all collected values from similarity analyses are displayed in Fig. S7.

Next, subsets of each size were split into two quantile bins for each sample quality metric separately. High sample quality bins (low average freeze-thaw cycles or high average RIN) were compared to low sample quality bins. High sample quality subsets were tested for higher similarity than low sample quality subsets using a one-sided Mann-Whitney U test.

Additionally, three linear regressions were fit to quantify the contribution of sample quality metrics to the change in similarity for DE results across subsets. We fit one model to predict similarity from freeze-thaw and RIN, while also accounting for the improvement in reproducibility due to increase in subset size (Similarity ~ Freeze-Thaw + RIN + Subset Size). We also fit two models predicting similarity from freeze-thaw or RIN alone.

### Discordance to assess differential expression reproducibility

We adapted a measure of concordance to measure discordance, or the lack of reproducibility, between differential expression results [55]. Average RIN and freeze-thaw were calculated for each subset (Fig. S8-9). Subsets for each subset size were split into two quantile bins for either sample quality metric (number of freeze-thaw cycles and RIN). Genes with a median base mean across all subsets in the bottom tenth percentile were excluded from the analysis (Fig. S5).

We do not use the original concordance at the top (CAT) metric because we are not comparing our results to a gold standard dataset. Instead, we use gene-wise LFC standard deviation across subsets as a measure of discordance. Thus, the average LFC for each gene across DE runs is analogous to the gold standard, and the dispersion from this average indicates a lack of reproducibility. At each combination of subset size and sample quality bins, we calculate discordance and compare it to the gene-wise median effect size (Fig. S8). We measure effect size as the mean-variance standardized effect [1]. This and two additional effect size metrics (Cohen’s d and absolute median LFC) we use are further described in Fig. S9. Results for all three effect size metrics reflect similar trends and can be found in Supplementary Tables S6-7.

We used a linear regression to predict discordance from effect size at each subset size. Additionally, in a separate linear regression, we account for the interaction between effect size and sample quality (Discordance ~ Effect Size x Sample Quality) at each subset size. Here, sample quality is a dummy variable, assuming a value of 0 for low quality and 1 for high quality. We did not include a term for subset size because regressions were fit within each subset size.

### Read coverage bias

The distribution of read coverage over each gene body was measured using *geneBody_coverage.py* from the *RSeQC* (v3.0.0) package [56]. We measure this coverage ranging from the 0th percentile (5′ end) to the 100th percentile (3′ end) nucleotide. The i^th^ percentile nucleotide is calculated as nucleotide_i_/length_gene_. Coverage at the i^th^ percentile nucleotide is normalized across all genes within a sample.

For a given sample, the median coverage percentile is defined as the nucleotide percentile at which median cumulative coverage is achieved; cumulative coverage is aggregated from the 5′ end to the 3′ end. The larger the median coverage percentile value, the larger the 3′ bias in coverage. We include 9 industry standards in our analysis--six of which had undergone five freeze-thaw cycles and three of which had undergone one freeze-thaw cycle--to explore the impact at higher freeze-thaw counts. We also include ribosomal RNA depleted samples as a negative control.

We conducted a meta-analysis of read coverage bias on ten publicly available blood tissue RNA-seq datasets. These datasets were either queried from SRA using pysradb (v0.11.1) [57] or manually identified. Altogether, these datasets contained samples that underwent both library preparation methods (poly(A)-enrichment and ribosomal depletion) and both sample handling conditions (frozen and unfrozen). We further verified queried datasets for accuracy of relevant conditions (e.g., tissue-type, sample handling) by manually checking the methods sections of associated publications. For the meta-analysis, SAM files were directly downloaded using the sam-dump command from SRA-toolkit (v2.8.2). SAM files were converted to bam, sorted, and indexed using SAMtools (v1.7). Gene body coverage was calculated from alignments using RSeQC as previously described. We also analyzed an additional dataset (phs000676.v1.p1), which contains frozen tissue samples from the UNC and TCG tumor tissue repositories [34]. We did not directly analyze the raw files from TCGA or UNC, but instead reanalyzed the reported 5′ to 3′ bias ratios. Conceptually, the smaller this ratio is, the larger the 3′ bias in read coverage.

## Supplementary Information

**Additional file 1.**

**Additional file 2.**

## Data Availability

The datasets supporting the conclusions of this article are available in Gene Expression Omnibus (GSE150097, https://www.ncbi.nlm.nih.gov/geo/query/acc.cgi?acc=GSE150097). Associated metadata is summarized in Supplementary Tables S2-3.
